# Fast and Sensitive Determination of Cadmium and Selenium in Rice by Direct Sampling Electrothermal Vaporization Inductively Coupled Plasma Mass Spectrometry

**DOI:** 10.3390/molecules27238176

**Published:** 2022-11-24

**Authors:** Guanyu Lan, Xue Li, Hongyu Jia, Xiaofeng Yu, Zhaohui Wang, Jijun Yao, Xuefei Mao

**Affiliations:** 1 Key Lab of National Soybean Industry Technology System, School of Food Science and Engineering, Jilin Agricultural University, Changchun 130118, China; 2 Institute of Quality Standard & Testing Technology for Agro-Products, Chinese Academy of Agricultural Sciences, Key Laboratory of Agro-Food Safety and Quality, Ministry of Agriculture and Rural Affairs, Beijing 100081, China; 3 Hangzhou Puyu Technology Co., Ltd., Hangzhou 311300, China

**Keywords:** cadmium, selenium, electrothermal vaporization, graphite furnace, bypass gas, inductively coupled plasma mass spectrometry

## Abstract

In this work, a direct solid sampling device based on modified graphite furnace electrothermal vaporization (GF-ETV) with inductively coupled plasma mass spectrometry (ICP-MS) was established for the simultaneous detection of trace selenium and cadmium in rice samples. A bypass gas was first designed in GF-ETV to improve the device’s analytical sensitivity and precision. The ashing and vaporization conditions, the flow rates of the Ar carrier and the bypass gases of ICP-MS were all investigated. Under the optimized conditions, the limits of detection (LODs) for Se and Cd were 0.5 μg kg^−1^ and 0.16 μg kg^−1^, respectively; the relative standard deviations (RSDs) of repeated measurements were within 8% (*n* = 6). The recoveries of Cd and Se in rice samples were in the range of 89–112% compared with the microwave digestion ICP-MS method, indicating good accuracy and precision for the simultaneous detection of Se and Cd in rice matrix. The whole analysis time is <3 min without the sample digestion process, fulfilling the fast detection of Se and Cd in rice samples to protect food safety.

## 1. Introduction

Selenium, widely existing in the human body, animals and plants, is well known for its health roles, such as anti-aging, inhibiting cardiovascular disease and cancer, and enhancing immune function. On the other hand, a long-term selenium deficiency might result in human diseases, including Ke-shan or Kaschin-beck diseases [1]. However, selenium is significantly maldistributed in China, and endemic selenium deficiency has been found in northeast and southwest China [2,3], while selenium is rich in the environmental medium of Anhui, Shandong, Hubei, and Shaanxi provinces [4,5,6]. It must be noticed that cadmium, as one kind of toxic heavy metal, is always associated with selenium-rich soil due to the parent materials of soil [7]. Associated with intaking selenium-rich food, cadmium is also ingested and accumulated in the human body, possibly leading to renal tubular dysfunction, neurotoxicity, osteoporosis, and even carcinogenic effects [8]. As the most important staple grain and selenium-rich food, rice is also considered to be one of the primary sources of dietary exposure to cadmium [9]. Therefore, a simultaneous and accurate analysis method for cadmium and selenium is indispensable for monitoring rice samples.

At present, the analyses of cadmium and selenium are frequently performed using conventional atomic spectrometric or inorganic mass spectrometric methods, such as inductively coupled plasma mass spectrometry (ICP-MS) [10,11], inductively coupled plasma optical emission spectrometry (ICP-OES) [12], atomic absorption spectrometry (AAS) [13], atomic fluorescence spectrometry (AFS) [14], etc. Among them, ICP-MS is characterized by high sensitivity, a wide dynamic linear range, and multi-elemental analysis, which is especially suitable for the simultaneous detection of ultra-trace Cd and Se. However, the ICP-MS methods are mainly reported on liquid sampling mode using a concentrically pneumatic nebulizer, thereby depending on a complicated sample digestion process with disadvantages such as time consumption (several hours and even two days), environmental unfriendliness (strong acid), and elemental contamination or loss. Thus, if canceling the digestion process and introducing solid samples into ICP-MS, the direct sampling analysis of Cd and Se will be fulfilled with the enhancement of sampling ratio and time efficiency.

In recent years, electrothermal vaporization (ETV), laser ablation ICP-MS, and X-ray fluorescence spectrometry (XRF) have been frequently reported as direct sampling analytical methods for Se, Cd and other elements in food samples. However, precise quantification is still a puzzle for LA-ICP-MS; the detection limits (LODs) of XRF are not available for trace Se and Cd in food samples [15,16]. So, ETV, usually made from graphite, high-melting metals or ceramic materials, is a feasible and versatile approach to solid sample introduction. Among them, graphite furnace was one of the most commonly used ETV setups, coupling with ICP-MS, ICP-OES, AAS or AFS via solid or slurry sampling; further, ETV-ICP-MS is an effective approach for the simultaneous detection of Cd and Se with the highest sensitivity and isotopic information. For instance, Li et al. determined Cr, Cu, Cd, Hg and Pb in rice samples using ultrasonic slurry sampling (USS)-ETV-dynamic reaction cell (DRC)-ICP-MS. Herein, ascorbic acid was selected as a modifier, and HNO_3_ and Triton X-100 were added, showing significant improvement in the ion signal; in addition, NH_3_ was used as a reaction gas in the DRC to alleviate the effect of interfering ions on the elements to be measured. Standard addition and isotope dilution methods were available for the calibration strategy, and the limits of detection (LODs) reached 0.4–1.7 ng·g^−1^ [17]. Zhang and Mao et al. used a tungsten coil trap (TCT) coupled with ICP-MS following solid sampling ETV device for the determination of Zn and Cd in agricultural products, of which on-line ashing and TCT effectively improved the elemental peak shape and memory effects, while was not feasible for Se. However, direct solid sampling of rice is easy to burn and splash in O_2_-containing atmospheres to indirectly impact the analysis accuracy, while slurry sampling easily avoids this [18,19]. Grindlay et al. determined ultra-trace Pb, Se, and As in wine using ETV-ICP-MS, again using ascorbic acid as a modifier. It was shown that excessive sample size resulted in excess matrix interferent. [20] To the best of our knowledge, the simultaneous detection of Cd and Se based on solid sampling ETV-ICP-MS has not been reported thus far.

When selenium and cadmium are detected by ICP-MS, they are susceptible to interference by double-charged ions, interfering elements such as Zn and Ca, and some oxides such as MoO and NbO. The development of multiple reaction modes for ICP-MS/MS is very effective for eliminating interference [21,22,23,24]; however, this does not work well for ETV mainly due to the short appearance time of elemental signal peaks after a fast vaporization process. Furthermore, the interference of moisture and organic substances can be removed via the drying and ashing process [17]. However, agricultural products contain high concentrations of components such as fat, protein and starch. In the direct sampling of solids, complex organic matrices can produce contamination in the ETV ashing procedure as well as the “carry-on effect” and “residual effect” caused by microparticles impacting the aerosol transport efficiency and even ionization efficiency [25]. Chemical modifiers, including Pd/Mg salts [26,27], Triton X-100 [28], ascorbic acid [29], and fluorinating agents [30,31,32], are often used in ETV applications to ameliorate the problem of matrix interference. However, when directly sampling solids, most chemical modifiers are of very limited use because of difficulties in adequate mixing and contact. This problem can be well solved when using slurry sampling with another advantage of easy calibration (matrix matching or standard addition) and automatic operation [33,34].

To accomplish the simultaneous detection of Cd and Se in rice samples, a graphite furnace-based ETV-ICP-MS for slurry sampling analysis was developed. The gas system, temperature control system, and tandem interface of the ETV unit were designed and optimized to meet the usage requirements of rice samples. A bypass gas line was first designed to remove residual matrices to improve the analytical sensitivity and stability. The proposed method has the advantages of being fast, efficient, and accurate for the simultaneous detection of Cd and Se in rice samples.

## 2. Results and Discussion

### 2.1. Design of Gas Line System

Solid sampling ETV was the earliest method that originating from the graphite furnace for AAS detection in the 1950s, which has become the most commonly used ETV approach at present. The ICP-MS hyphenation with ETV has also been reported from the 1990s and was utilized for the detection of Cd or Se separately or with other elements. However, the conventional graphite furnace for AAS is not specially designed for ICP-MS, so the gas line and analyte transportation must be modified and improved for the hyphenation of ETV and ICP-MS.

**Figure 1:**
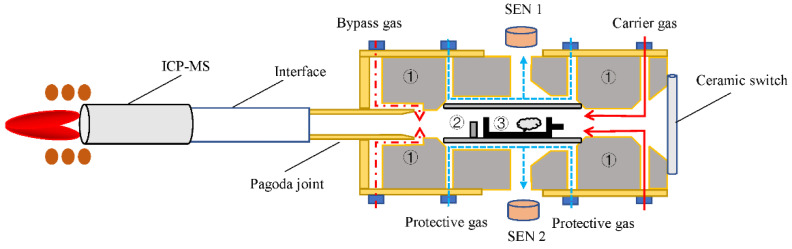
Schematic diagram of DS-ETV-ICP-MS instrumentation. SEN1 and SEN2 refer to the photoelectric sensor and infrared sensor, respectively. ① refers to the graphite electrode embedded in the copper base. ② and ③ refer to the graphite tube and sample boat, respectively. The red solid line refers to the carrier gas path, the blue dotted line refers to the protective gas flow path and the red dotted line refers to the bypass gas flow path, respectively.

For the conventional graphite furnace for AAS, only the carrier gas line was designed for analyte transportation to ICP-MS. However, when a graphite furnace mentioned above was employed, elemental and matrices deposition was found on the outlet of the graphite furnace (Figure S1) after 50 repeated measurements, thereby leading to the loss of sample introduction efficiency. This might be due to the “dead area” caused by the wind-resistance structure in the channel and the “cold area” vs. the heating area in the furnace, in which vaporized aerosols are more readily condensed on the inner surface under lower temperatures [35]. So, we designed a bypass gas line with annular and symmetrical tetra-holes downstream of the graphite furnace outlet, as shown in Figure 1. When carrier gas took analytes vaporized out of the graphite furnace, bypass gas mixed with carrier gas to accelerate forming turbulence and speed up the transportation of aerosol containing Cd and Se; at this moment, matrices deposition mentioned above can be effectively relieved as shown in Figure S1, to thereby avoid the memory effect.

**Figure 2:**
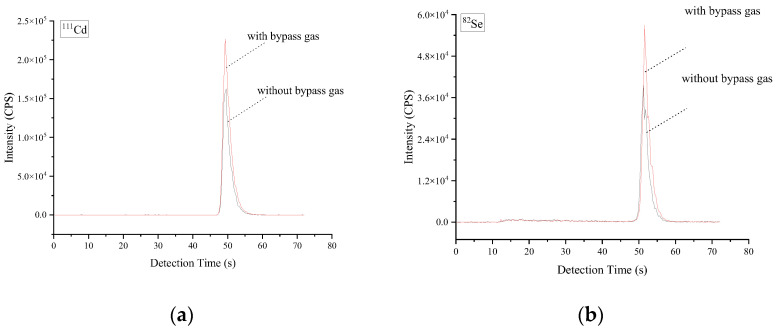
The effect of the gas line design with or without bypass gas on ICP-MS signals of Cd and Se. The other operating parameters are consistent with Table 3. A rice sample (Cd = 19 ± 1 ng·g^−1^ and Se = 36 ± 2 ng·g^−1^) was employed for slurry sampling. (**a**) Cd signal peak with or without bypass gas. (**b**) Se signal peak with or without bypass gas.

On the other hand, the effect of the gas line design with or without bypass gas on ICP-MS signals of Cd and Se was investigated using a slurry rice sample under 600 mL·min^−1^ bypass argon, and the results are shown in Figure 2. By comparison, the bypass gas enhanced the ICP-MS signal intensities (peak area) of Cd and Se by ~33% and ~32%, respectively, vs. no bypass gas design. This result might be caused by the fact that the bypass and carrier gases mixture makes gaseous analytes sufficiently turbulent to collide with each other to form a stable aerosol-like “atomic cluster” in the previous literature [35,36]. In addition, the signal loss of elemental deposition can be avoided as much as possible. As a result, the bypass gas line design was retained for the following study.

### 2.2. Sample Dehydration and Ashing

**Figure 3:**
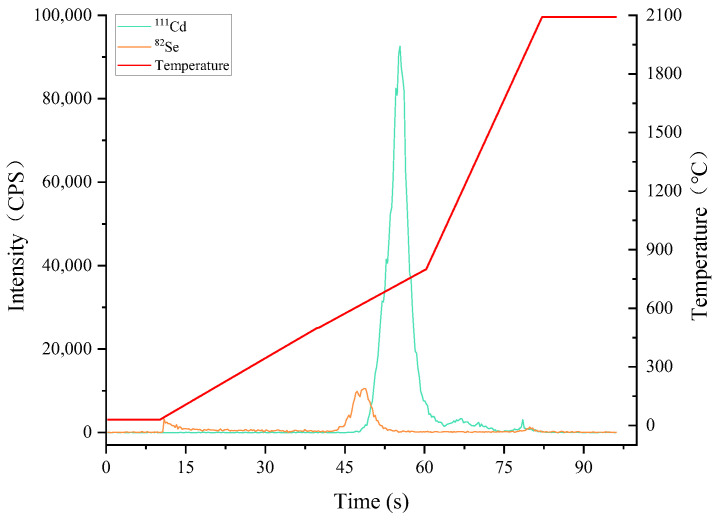
Cd and Se signal peaks with the increase of ETV heating time (temperature). The operating parameters of ETV monitoring are shown in Table S3. A rice sample (GBW10010a, Cd = 53 ± 4 ng·g^−1^ and Se = 36 ± 8 ng·g^−1^) was employed for slurry sampling.

Due to the presence of starch, protein, and water in the rice matrix, rice samples must be dehydrated and ashed to remove these prior to Cd and Se vaporization to eliminate the matrix interference, as well as to avoid the loss of Cd and Se analytes. Therefore, the dehydrating and ashing temperatures were investigated, and the results using a rice sample are shown in Figure 3. With the increase of heating time, the temperature went up, of which Cd and Se signal peaks appeared from 550 °C and 620 °C, respectively. To avoid Se loss, the ashing temperature must be set below 550 °C, and 450 °C was proved effective for rice sample ashing via observation. At the same time, to avoid the bumping of rice slurry (analyte loss), the dehydration process was carried out under a gentle heating speed; as a result, room temperature to 200 °C within 25 s and then holding for 15 s was chosen. Then, the ashing process was performed from 200 °C to 450 °C within 20 s and then held for 20 s. To prevent matrix interferents entering the following ICP-MS, carrier (300 mL·min^−1^) and bypass (100 mL·min^−1^) argon gases were employed to blow the moisture and organic substances outside via the switching outlet.

### 2.3. Vaporization of Cd and Se

After the ashing process, Cd and Se remain in the sample residue; and they should be continuously heated to fulfill the complete vaporization from the residual. From Figure 3, Cd and Se can be completely vaporized below 1000 °C, which is consistent with the previous study [8]; furthermore, no signal peaks of Cd and Se were found from 1000 °C to 2100 °C. In order to minimize the memory effect, the final vaporization temperature was set at 1900 °C.

**Figure 4:**
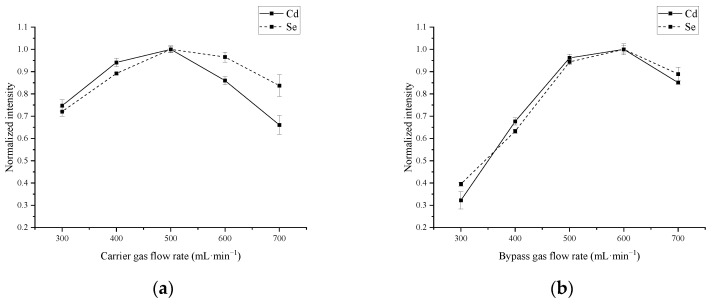
The effect of carrier and bypass gases on Cd and Se signal intensities. The other operational parameters are consistent with Table 3. A rice sample (GBW 10045a rice sample, Cd = 320 ± 40 ng g^−1^ and Se = 60 ± 1 ng g^−1^) was employed for slurry sampling. (**a**) The effect of carrier gas on Cd and Se signal intensities. (**b**) The effect of bypass gas on Cd and Se signal intensities.

In addition, the gas atmosphere is also a crucial factor for the vaporization of Cd and Se. Therefore, the effect of carrier and bypass gases was investigated, and the results are shown in Figure 4. The highest signal intensities of Cd and Se were found under 500 mL min^−1^ carrier gas and 600 mL min^−1^ bypass gas with favorable RSDs (1.2–1.5%), respectively. Prior to the signal summits, the higher flow rates of carrier and bypass gases mixture facilitate the transportation of Cd and Se analytes, while too-high flow rates result in a dilution effect to reduce the ICP-MS signal intensities. Finally, the carrier and bypass gases were set as 500 mL min^−1^ and 600 mL min^−1^, respectively.

### 2.4. Interference Study

To further investigate the anti-interference capacity of this proposed method, Cd and Se standard solutions were detected by ETV-ICP-MS using adding different chemicals such as 1% of K_2_Cr_2_O_7_, urea, and NH_4_H_2_PO_4_, and 10 mg/L of Fe, As, Na, Ca, Mg and Pb into them, and the results are shown in Table 1. Herein, the recoveries of 92–116% for Se and 88–120% for Cd indicate the absence of significant interference caused by these potential substances.

However, the systemic errors of this ETV-ICP-MS method caused by the rice sample matrix still remain, in which the signal intensities of rice samples with the same Cd and Se levels were obviously higher than that of standards in Figure S2. This result was consistent with other studies reported previously [37,38]. Therefore, the matrix effect cannot be ignored, and the curve slopes of standard addition calibrations (using rice slurry mixed with different Cd and Se standard solutions) were obviously higher than those of the standard solution calibrations in Figure S3. As a result, to calibrate the analysis of Cd and Se in rice samples, the calibration strategy of the standard addition method was utilized for the following quantitative analysis.

### 2.5. Analytical Performances and Rice Sample Analysis

Under the optimized conditions, the method limits of detections (LODs) were calculated using the formula 3 *σ/m* with 11 measurements of Cd or Se in 10 μL Triton-100 (1:5000, *v*:*v*) (*σ* is the standard deviation and *m* is the slope of the calibration graph of Cd or Zn), and they were 5 fg for Cd and 1.6 fg for Se, namely 0.5 μg/kg and 0.16 μg/kg using 10 μL rice slurry sample, respectively. The linear range (*R*^2^ > 0.999) using the standard addition method ranges from 0.001 to 1 ng, corresponding to 1–1000 ng g^−1^ of Cd and Se in rice samples, respectively.

To verify the accuracy and precision, rice samples, including CRMs, were analyzed (*n* = 6) using this proposed method and the microwave digestion ICP-MS method. As shown in Table 2, the recoveries are from 89% to 112% in the rice samples and adequate agreement was observed with the certified values of the CRMs (GBW10010a and GBW10045a with certified 53 ± 4, 320 ± 40 ng g^−1^ Cd and 36 ± 8, 60 ± 10 ng g^−1^ Se values, respectively) and the results of microwave digestion ICP-MS. The RSDs of the repeated measurements (*n* = 6) of Cd and Se ranged from 0.4% to 7.7%, indicating favorable analytical accuracy and feasibility. The whole analysis of Cd and Se in rice samples can be finished within 3 min without the complicated sample digestion process, proving a rapid, green, and robust analytical instrumentation.

## 3. Materials and Methods

### 3.1. Instrumentation

The direct sampling electrothermal vaporizer (DS-ETV) was made in our laboratory, and its schematic diagram is shown in Figure 1. The actual device picture is displayed in Figure S4. The DS-ETV system mainly consists of a graphite furnace, an automatic sampling device (EXPEC 723, Hangzhou PuYu Technology Development Co., Ltd., Hangzhou, China, 40 positions) with graphite boats, a temperature sensor and control system, a gas line system, an interfacing tube for ICP-MS, and a power supply. Herein, the graphite furnace (28 × 7.4 mm, Φ 5.9 mm, Beijing Xiangchenghe Photoelectric Technology Co., Beijing, China) and sample boat (18 × 4.2 × 3.3 mm, 120 μL, Beijing Xiangchenghe Photoelectric Technology Co., Beijing, China) were both coated with pyrolyzed carbon (methane and ammonia at high temperature to form a pyrolytic coating according to the previous study [39]) were employed to heat (longitudinal heating with graphite electrodes) a slurry aliquot of powdered rice. To monitor the real-time temperature of the graphite furnace, a photoelectric sensor (S1226-44BQ, Beijing Hamamatsu Photon Techniques Inc., Beijing, China) and an infrared thermometer (SA120BSK, Wuxi Shiao Technology Co., Ltd., Wuxi, China) were jointly utilized, and then to control the heating temperature ranging from 0 to 2600 °C via a proportional integral differential (PID) based on a computer and a power supply (AC, 50 Hz). The gas line system is mainly composed of two gas mass flow controllers (GMFC) and a needle valve to control carrier gas, bypass gas, and protective gas, respectively. A cyclonic nebulizer was made of PFA (inner capacity ~40 mL) and interfaced with the ETV outlet with ICP-MS (SUPEC 7000, Hangzhou PuYu Technology Development Co., Ltd., Hangzhou, China). The detailed parameters of DS-ETV-ICP-MS instrumentation are shown in Table 3 and Table 4.

To verify the proposed method, a separate ICP-MS was used to detect Cd and Se in rice samples after the microwave digestion process. The detailed operating conditions of microwave digestion (EXPEC 790S, Hangzhou PuYu Technology Development Co., Ltd., Hangzhou, China) and ICP-MS (SUPEC 7000, Hangzhou PuYu Technology Development Co., Ltd., Hangzhou, China) are listed in Table S1.

### 3.2. Chemicals and Standards

The standard stock solutions (1000 mg L^−1^) of Cd, Se, and other elements were purchased from the National Center for Analysis and Testing of Nonferrous Metals and Electronic Materials (Shanghai, China). The certified reference materials (CRMs) of powered rice (GBW10010a and GBW10045a with certified 53 ± 4, 320 ± 40 ng g^−1^ Cd and 36 ± 8, 60 ± 10 ng g^−1^ Se values, respectively) were purchased from the National Research Center for Certified Reference Materials (Beijing, China). Working standards were obtained by stepwise dilution of stock solutions with deionized (DI) water, which was prepared using a Milli-Q Elix Essential water purification system (Millipore, Burlington, MA, USA). HNO_3_ and H_2_O_2_ (guaranteed grade) were used for the microwave digestion of rice samples. Triton X-100 from PerkinElmer (Waltham, MA, USA) was used to prepare slurry samples of powdered rice after sieving by 60-mesh.

### 3.3. Analytical Procedures of Direct Sampling ETV-ICP-MS

The analytical procedures of slurry direct sampling ETV-ICP-MS for Cd and Se in rice sample are summarized as follows: (1) dehydration: a 10 μL slurry sample was pipetted into the sampling boat and then was automatically moved into the graphite furnace for heating under 200 °C for 15 s to complete the dehydration. Here, carrier and bypass argon gases were set as 300 mL min^−1^ and 100 mL min^−1^, respectively, to blow the moisture outside via the switching outlet. (2) Ashing: the graphite furnace was heated to 450 °C within 20 s and held for 20 s to ash the rice sample; the above carrier and bypass gases were employed to blow the organic substance outside via the switching outlet. (3) Vaporization: the graphite furnace was heated to 1900 °C within 7 s and then kept for 5 s. The carrier and bypass argon gases were changed to 600 mL min^−1^ and 500 mL min^−1^, respectively, when switching off the outlet mentioned above. (4) Detection: the Cd and Se analytes were transported into the ICP to excite them for the following detection using a quadrupole mass spectrometer, of which peak area (cps) was calculated for the quantitative analysis. (5) Cleaning: 500 mL min^−1^ carrier Ar and 800 mL min^−1^ bypass Ar under 2100 °C was performed for 5 s to eliminate the potential interference caused by residual matrix in the furnace and transportation.

### 3.4. Analytical Procedures of Microwave Digestion ICP-MS

A 0.1 g rice sample was accurately weighed and placed in a modified tetrafluoroethylene (TFM) digestion vessel with 1 mL HNO_3_, 2 mL H_2_O_2_, and 1 mL DI water. The procedures of the microwave digestion system (EXPEC 790S, Hangzhou PuYu Technology Development Co., Ltd., Hangzhou, China) are shown in Table S1. After microwave digestion, the sample solutions were cooled to 40 °C and then transferred to a 50 mL centrifuge tube to be diluted to 15 mL with 2% HNO_3_ (*v*:*v*). The digested solutions will be measured by ICP-MS, as shown in Table S2.

### 3.5. Sample Preparation

Rice samples were purchased online. The rice samples were first dried at 70 °C for 4 h in a constant temperature oven (101-3B, Union Whale Electronic Technology Co., Ltd., Shanghai, China). The dried samples were ground using a grinder (BJ-800A, Deqing Baijie Electric Co., Ltd., Huzhou, China) and then passed through a 60-mesh sieve. According to the previous studies [40,41], 0.1 g rice powder was placed into a crushing tube (pre-placed zirconium beads, Φ 0.15 mm, 1.5 g) and 1 mL of 1:5000 (*v*:*v*), and then Triton-100 solution was added. After sealing, the sample was placed in a grinder (DS1000, Hubei Xinzongke Viral Disease Engineering Technology Co., Ltd., Wuhan, China) and ground 3 times at 5500 rpm for 15 s. After grinding, the tube was placed in a shaker (NMYJ-12, Taizhou Nomi Medical Technology Co., Ltd., Taizhou, China) and shaken for 120 s. The above sample preparation can be finished within 7 min.

### 3.6. Statistical Analysis

Statistical analysis of experimental data was performed using Origin 2021 (OriginPro 2021 9.8.0.200, OriginLab Co., Northampton, Massachusetts, USA) and Microsoft Excel^R^ 2019 (2210 Build 16.0.15726.20070, Microsoft Co., Washington, Redmond, USA). Significant differences were assessed by a *t*-test, in which *p*-values lower than 0.05 (*p* < 0.05) supported statistical significance.

## 4. Conclusions

In this work, a novel solid sampling Cd-Se analyzer was for the first time developed for the direct and simultaneous determination of trace Se and Cd in rice samples. The bypass gas line was newly designed to improve analytical sensitivity and precision. Thus, the method LOD of Se and Cd reached 0.5 μg kg^−1^ and 0.16 μg kg^−1^, respectively, for 10 μL rice slurry sample. For the novel DS-ETV-ICP-MS instrumentation, the whole analysis time was <3 min without the sample digestion process. Thus, the proposed direct sampling method has a promising application potential in the rapid, sensitive, green and robust detection of multi-elements in food samples.

## Figures and Tables

**Table 1:** The interference of potential substances (*n* = 3).

Substances	Added Cd ^a^ (ng)	Measured Cd by the Proposed Method (ng)	Recovery ^b^ (%)	Added Se ^a^ (ng)	Measured Se by the Proposed Method (ng)	Recovery ^b^ (%)
K_2_Cr_2_O_7_	0.2	0.176 ± 0.003	88	0.2	0.187 ± 0.003	93
Urea	0.2	0.239 ± 0.009	120	0.2	0.185 ± 0.004	92
NH_4_H_2_PO_4_	0.2	0.197 ± 0.020	99	0.2	0.232 ± 0.016	116
Fe	0.2	0.204 ± 0.013	102	0.2	0.200 ± 0.001	100
Mg	0.2	0.179 ± 0.007	90	0.2	0.193 ± 0.002	97
Na	0.2	0.189 ± 0.010	95	0.2	0.192 ± 0.002	96
Ca	0.2	0.206 ± 0.011	103	0.2	0.198 ± 0.009	99
As	0.2	0.191 ± 0.009	95	0.2	0.198 ± 0.004	99
Pb	0.2	0.222 ± 0.009	111	0.2	0.214 ± 0.011	107

^a^ A series of 10 μL of 20 ng·mL^−1^ Cd and Se standard solutions containing several substances at the above-added levels were measured using the proposed method. ^b^ Recovery (%): Cd or Se (ng) measured by the proposed method/added Cd or Se (ng)*100%.

**Table 2:** Cd and Se presence and recoveries in rice samples (*n* = 6).

Samples ^a^	Cd				Se			
	ICP-MS or Certified (ng·g^−1^)	This Method (ng·g^−1^)	RSD (%)	Recovery ^b^ (%)	ICP-MS or Certified (ng·g^−1^)	This Method (ng·g^−1^)	RSD (%)	Recovery ^b^ (%)
THX	169 ± 2	174 ± 2	1.0	103	50 ± 4	53 ± 1	1.0	105
JS	149 ± 1	151 ± 2	1.6	101	126 ± 4	123 ± 2	1.7	98
ZS	19 ± 1	20 ± 1	4.0	106	36 ± 2	36 ± 2	4.7	100
QY	134 ± 1	132 ± 4	3.1	98	33 ± 3	31 ± 1	3.6	93
HN-1	419 ± 2	421 ± 5	1.3	100	25 ± 3	26 ± 1	5.3	105
HN-2	213 ± 1	223 ± 1	0.4	105	27 ± 2	26 ± 1	4.3	98
FZ	4 ± 1	4 ± 1	7.7	100	17 ± 2	17 ± 1	4.3	100
GBW10010a	53 ± 4	47 ± 2	4.9	89	36 ± 8	40 ± 2	5.2	111
GBW10045a	320 ± 40	309 ± 5	1.5	97	60 ± 1	67 ± 2	2.7	112

^a^ For each sample, 10 μL of the prepared slurry sample was measured according to the proposed method. ^b^ The results of this method are compared to the certified values in Cd and Se concentrations of CRMs; or to the measured values by the microwave digestion ICP-MS method for the recovery calculation.

**Table 3:** Instrumental program of direct sampling ETV device.

Procedure	ETV Temperature (°C)	Heating Time (s)	Holding Time (s)	Carrier Gas Flow (mL min^−1^)	Bypass Gas Flow (mL min^−1^)	Signal Acquisition
Dehydration	200	25	15	300	100	
Ashing	450	20	20	300	100	
Vaporization	1900	7	5	500	600	Yes
Detection						
Cleaning	2100	5	5	800	500	

**Table 4:** Instrumental program of ICP-MS for direct sampling ETV.

Instrument Parameters	Setting Value
RF power/(W)	1400
Auxiliary flow/(L min^−1^)	1
Cooling gas flow/(L min^−1^)	14
Sampling depth/(mm)	2
Sampling cone/interception cone	Ni/Cu
Scanning times	1
Scan mode	peak hopping scan
Dwell time/(ms)	10
Isotopes	^111^Cd, ^82^Se

## Data Availability

The data presented in this study are available on request from the corresponding author. The data are not publicly available due to the privacy of the study participants.

## Supplementary Materials

The following supporting information can be downloaded at: https://www.mdpi.com/article/10.3390/molecules27238176/s1, Figure S1: Effect of bypass gas on residual deposition on the pagoda joint; Figure S2: The signal intensities of Cd and Se in real rice samples vs. standard solution; Figure S3: The calibration curves of Cd and Se with standard addition vs. standard solution; Figure S4: Picture of DS-ETV-ICP-MS instrumentation; Table S1: Microwave digestion program; Table S2: Instrumental conditions and parameters of ICP-MS; Table S3: Heating program of DS-ETV for Cd and Se; Table S4: Sample information of rice.
