# Supplementary material for: Fast and Sensitive Determination of Cadmium and Selenium in Rice by Direct Sampling Electrothermal Vaporization Inductively Coupled Plasma Mass Spectrometry
Source: Molecules. 2022 Nov 24;27(23):8176. doi: 10.3390/molecules27238176 (PMC9739379; doi:10.3390/molecules27238176)
Supplement: Supplementary file 1 [file molecules-27-08176-s001.zip › molecules-2034418-supplementary.pdf]

Supporting Materials for

Fast and sensitive determination of cadmium and selenium in rice by direct sampling electrothermal vaporization inductively coupled plasma mass spectrometry

Guanyu Lan [1,2,3 †], Xue Li [2,†], Hongyu Jia [1,2], Xiaofeng Yu [3], Zhaohui Wang [1,*] Jijun Yao [3,*], and Xuefei Mao [2,*]

[1] Key Lab of National Soybean Industry Technology System, School of Food Science and Engineering, Jilin Agricultural University, Changchun 130118, China

[2] Institute of Quality Standard & Testing Technology for Agro-Products, Chinese Academy of Agricultural Sciences, Key Laboratory of Agro-food Safety and Quality, Ministry of Agriculture and Rural Affairs, Beijing 100081, China

[3] Hangzhou Puyu Technology Co. Ltd., Hangzhou, Zhejiang 311300, China

***** Correspondence: wzhlindsp@aliyun.com (Z.W.); jijun_yao@fpi-inc.com (J.Y.); mxf08@163.com or maoxuefei@caas.cn (X.M.)

† These authors contributed equally to this work.

Table S-1 Microwave digestion program

Program	Temperature (℃)	Holding time (min)
5	150	3
5	200	3
8	240	30

Table S-2 Instrumental conditions and parameters of ICP-MS

Instrument parameters	Setting value
RF power/ (W)	1400
Nebulization gas flow/ (L min^{-1})	1.180
Auxiliary gas flow/ (L min^{-1})	1.00
Cooling gas flow/ (L min^{-1})	14.00
Sampling depth/ (mm)	2
Sampling cone/interception cone	Ni/Cu
Scanning times	10
Dwell time/ (ms)	10
Scan mode	peak hopping scan
Collision gas	He
Collision gas flow / (mL min^{-1})	1.25
Isotope	^{111}Cd、^{82}Se
Internal standard	^{45}Sc、^{103}Rh、^{115}In、^{185}Re、^{209}Bi

Table S-3 Heating program of DS-ETV for Cd and Se

ID	ETV temperature (℃)	Heating time (s)	Holding time (s)	Carrier gas flow (mL min^{-1})	Bypass gas flow (mL min^{-1})	Reading
1	100	5	2	500	600	√
2	200	5	2	500	600	√
3	300	5	2	500	600	√

4	400	5	2	500	600	√
5	500	5	2	500	600	√
6	600	5	2	500	600	√
7	700	5	2	500	600	√
8	800	5	2	500	600	√
9	1200	5	2	500	600	√
10	1600	5	2	500	600	√
11	2000	5	2	500	600	√

Table S-4 Sample information of rice

Sample	Place of production	Variety
THX	Changsha, Hunan	*Indica* rice
JS	Hengyang, Hunan	*Japonica* rice
ZS	Enshi, Hubei	*Indica* rice
QY	Enshi, Hubei	*Indica* rice
HN-1	Changsha, Hunan	*Indica* rice
HN-2	Changsha, Hunan	*Japonica* rice
FZ	Harbin, Heilongjiang	*Japonica* rice

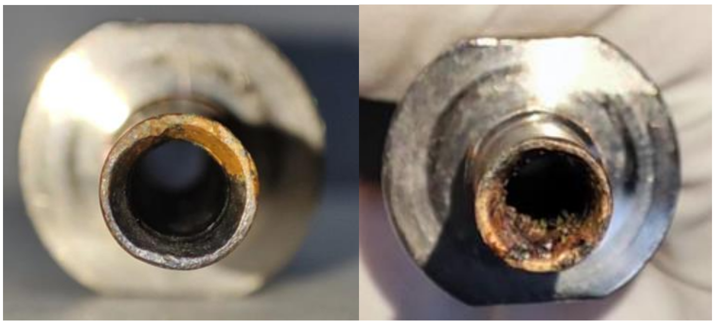

Figure S1 Effect of bypass gas on residual deposition on the pagoda joint. The picture on the left shows the pagoda joint with bypass gas after sampling 50 replicates, and the inner wall is smooth and there is no obvious residual deposition. The picture on the right shows the pagoda joint without bypass gas after sampling 50 replicates, and the residual deposition was obviously found.

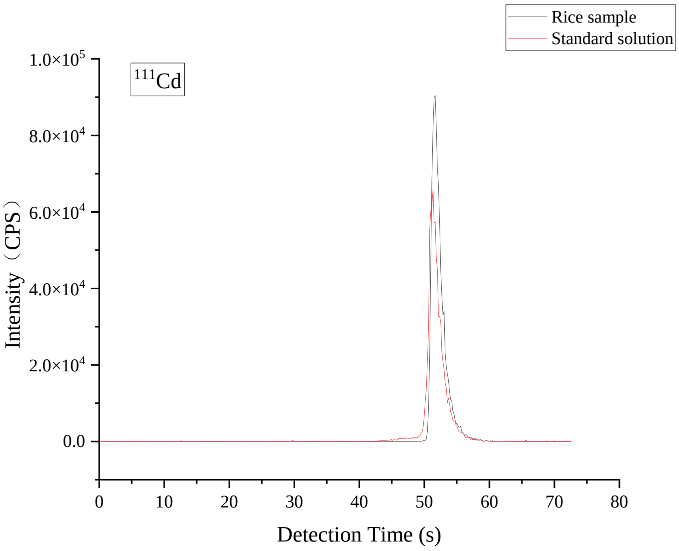

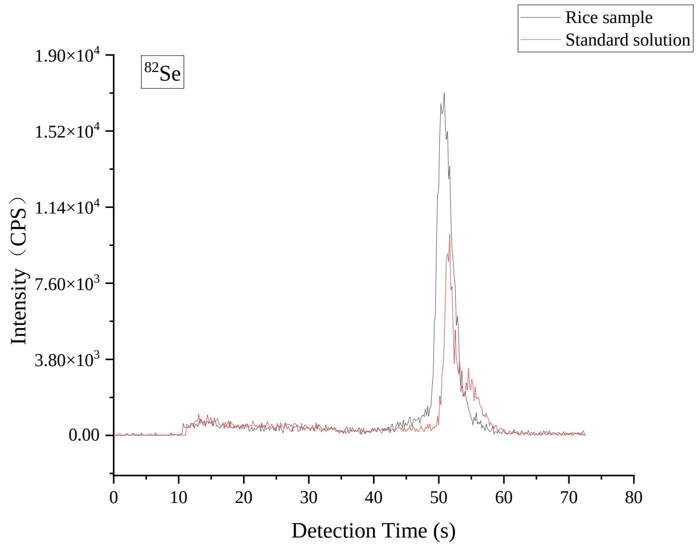

Figure S2 The signal intensities of Cd and Se in real rice samples *vs.* standard solution. The operational parameters were consistent with **Table 3**. For black line, a rice sample (GBW10010a, Cd = 53 ± 4 ng g^{-1} and Se = 36 ± 8 ng g^{-1}) was employed for slurry sampling. For red line, the rice sample was replaced with a standard solution of 53 ng L^{-1} Cd and 36 ng L^{-1} Se; and then 10 μL of slurry aliquot prepared was introduced into the ETV for measurement.

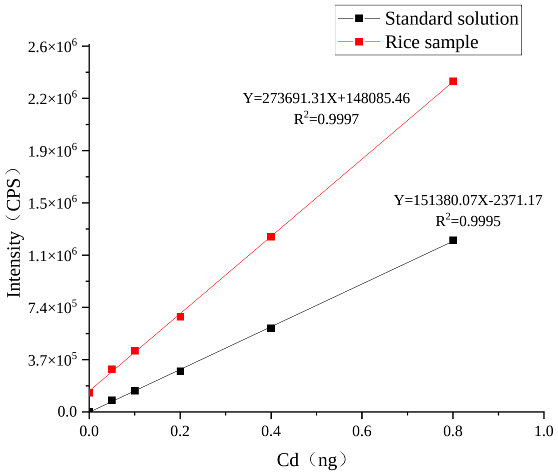

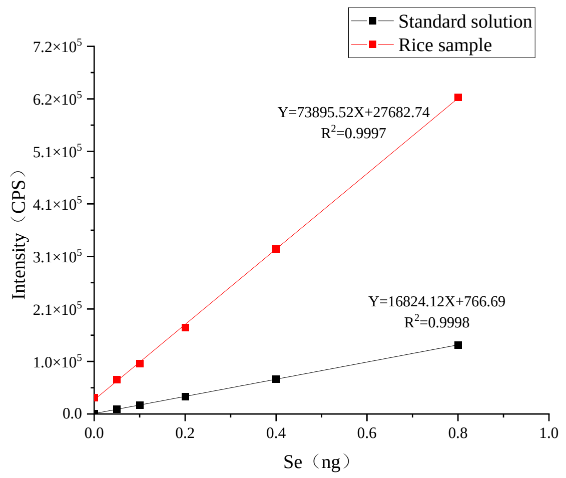

Figure S3 The calibration curves of Cd and Se with standard addition *vs.* standard solution. The operational parameters were consistent with **Table 3**. For the standard addition method, a rice sample (GBW10010a, Cd = 53 ± 4 ng g^{-1} and Se = 36 ± 8 ng g^{-1}) was employed for slurry sampling and spiked by 0.05, 0.1, 0.2, 0.4 and 0.8 ng of Cd or Se. For the standard solution method, 0.05, 0.1, 0.2, 0.4 and 0.8 ng Cd or Se were introduced into the ETV for measurement using 10 μL standard solution.

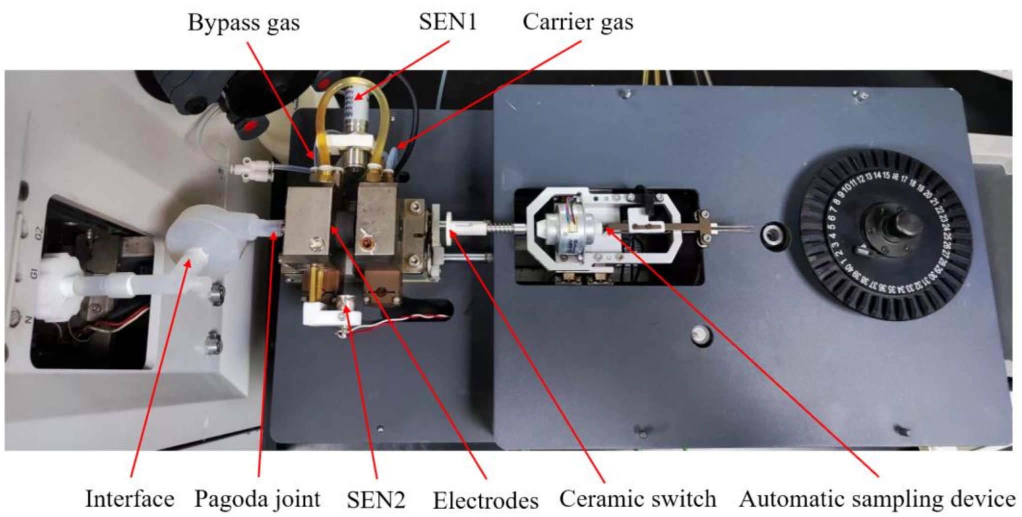

Figure S4 Picture of DS-ETV-ICP-MS instrumentation. SEN1 and SEN2 refer to the photoelectric sensor and infrared sensor, respectively. SSD refers to the signal delay device.
